# Is Abdominal Muscle Activity Different from Lumbar Muscle Activity during Four-Point Kneeling?

**Published:** 2013-12

**Authors:** Soraya Pirouzi, Farahnaz Emami, Shohreh Taghizadeh, Ali Ghanbari

**Affiliations:** 1 Center for Human Science Research, School of Rehabilitation Sciences, Shiraz University of Medical Sciences, Shiraz, Iran;; 2 Student Research Committee, Department of Physiotherapy, Chamran Hospital, Shiraz University of Medical Sciences, Shiraz, Iran;; 3 Department of Physiotherapy, School of Rehabilitation Sciences, Shiraz University of Medical Sciences, Shiraz, Iran

**Keywords:** Electromyography, Exercise therapy, Skeletal muscles

## Abstract

**Background: **Stabilization exercises can improve the performance of trunk and back muscles, which are effective in the prevention and treatment of low back pain. The four-point kneeling exercise is one of the most common types of stabilization exercises. This quasi-experimental study aimed to evaluate and compare the level of activation between abdominal and lumbar muscles in the different stages of the four-point kneeling exercise.

**Methods: **The present study was conducted on 30 healthy women between 20 and 30 years old. Muscle activity was recorded bilaterally from transversus abdominis, internal oblique, and multifidus muscles with an electromyography (EMG) device during the different stages of the four-point kneeling exercise. All the collected EMG data were normalized to the percentage of maximum voluntary isometric contraction. The repeated measures ANOVA and paired t-test were used for the statistical analysis of the data.

**Results:** A comparison between mean muscle activation in right arm extension and left leg extension showed that left internal oblique and left transverse abdominis muscles produced greater activation during left leg extension (P<0.05). The comparison of mean muscle activation between right arm extension and the bird-dog position showed that, except for the right internal oblique, all the muscles produced higher activation in the bird-dog stage (P<0.05). In comparison to the bird-dog stage, the left multifidus showed high activation during left leg extension (P<0.05).

**Conclusion: **The results of this study showed that the activity of all the above-mentioned muscles during quadruped exercise can provide stability, coordination, and smoothness of movements.

## Introduction

There are two important principles in the vertebral column: stability and mobility. Joint stability is defined as the effective adaptation of the joints to each specific load demand. Gravity, muscle, and ligament forces can produce joint compression, which leads to spinal stability in different conditions.^1^^-^^4^ Complex loading patterns impact the passive structures of the osteoligamentous spine and, if unprotected, the lumbar spine is rendered vulnerable to being impaired.^5^^,^^6^ The other main factor which affects stability is movement. Spinal stability is needed for appropriate movements,^1^^-^^4^ and movement can affect stability.

It has been estimated that 75-80% of the world population suffer from low back pain (LBP) at least once in their lives.^7^^,^^8^ Although rarely life-threatening, LBP is extremely disabling and indirectly impinges on national economies.^9^^,^^10^ Several studies have confirmed that the function and co-operation of the stabilizing muscles of the lumbar spine are often impaired in patients with LBP. Muscles play an important role in the etiology, presentation, and treatment of low back disorders. It is clinically known that excessive motion beyond the normal physiological limits, sometimes known as spinal instability, may cause chronic LBP.^11^^-^^13^ Recently, a large number of studies have shown that exercising can prevent, treat, and manage LBP. Stability exercises maintain the trunk stability because they restore the vertebral column against the perturbations of movement and activity in daily life.^14^^-^^16^

Up to now, a great deal of research has been conducted on the role of muscles in stabilizing the spinal column. For example, the relative activation amplitudes of oblique, transverse abdominis (TrA), and rectus abdominis muscles were evaluated during the pelvic tilt, abdominal hollowing, and level 1 of the trunk stability test exercises. The last one was used as an initial progression to assess the paravertebral musculature ability in the supine position (with hips and knees in 90° flexion while maintaining abdominal hollowing); it was concluded that muscle activation increased during these exercises.^15^ In 2007, Stevens and his colleagues^5^ noticed that the activation of internal oblique (IO), rectus abdominis, and multifidus muscles increased during the four-point kneeling position. Comparison between the local abdominal and back muscles demonstrated higher activation in local abdominal muscles in bird-dog and four-point kneeling exercises.^17^ Furthermore, the activation of rectus abdominis, TrA, and internal and external oblique muscles was investigated during abdominal hollowing in four different positions. The results suggested that all the four positions could facilitate the activation of TrA, IO, and rectus abdominis muscles, while external oblique muscles had minimal activation.^18^ Another study reported the increased activation of local and global muscles during the stabilization exercise on unstable surfaces.^19^

The appropriate type of exercise and the importance of the role of each muscle in these exercises have never been investigated. However, it has been suggested that the exercises which improve muscle stiffness should be encouraged in rehabilitation programs.^20^ Therefore, the present study aimed to compare the level of contraction between abdominal and lumbar muscles in order to clarify the role of the trunk (core) muscle activation during the four-point kneeling exercise. The effects of the motion of the upper and lower extremities on the trunk muscle activation were evaluated as well. 

## Materials and Methods

This quasi-experimental study was carried out in the Research Center of Shiraz Rehabilitation Department, Shiraz University of Medical Sciences, Shiraz, Iran. Considering a power of 0.8 with an alpha of 0.05, the sample size was calculated as 30 healthy subjects. The study population was, therefore, comprised of 30 healthy, right-handed women aged between 20 and 30 years with no known neuromuscular, orthopedic, or cardiovascular conditions. Also, the subjects had no previous experience of stabilization exercises. All the subjects signed written informed consents for participation in the study. Past recurrent LBP, Body Mass Index greater than 27, current neurological deficits, pain or disability of the upper or lower limbs, and left-handedness were the exclusion criteria. 

*Equipment*

The study data were collected using MegaWin software (Mega Electronics Ltd., Finland [v. 2.5 a 16]). Electromyography (EMG) signals were recorded using 6 pairs of self-adhesive disposable disc surface electrodes (Medico Lead-Lok) with an electrical contact of one cm² and a centre-to-centre distance of 2 cm. The electrodes were placed bilaterally over the muscle bellies of the TrA (as in the study conducted by Vezina),^15^ multifidus (as in the studies performed by Stevens, Won-gue, and Filho),^5^^,^^11^^,^^12^ and IO (as in the Stevens study).^5^ In addition, the skin was prepared and the skin resistance was decreased to lower than 5 kilo-ohms. Prior to processing the raw EMG data, a customized quality control program in conjunction with visual inspection was used on all the channels in order to detect and eliminate the possible contamination of the EMG signal by heartbeat and other artifacts. The EMG data were amplified and fully rectified with a band-pass filter at 5-500 Hz and then sampled at 1000 Hz. Thereafter, the data were recorded onto a hard disk and transferred to floppy disks for offline processing. 

The electrode sites were validated using manual muscle testing and doing maximal voluntary contraction (MVC) to isolate each instance of muscle activation and decrease cross-talk.^15^ Each channel had an isolated ground electrode in order to minimize the noise, and the electrodes were well taped in order to prevent the artifact. To ensure a stable temperature and impedance, no recording was made within 10 minutes of electrode placement. The subjects were asked to relax completely in the supine position, and the noise of the channels was kept at less than 5 kilo-ohms.

*Data Collection*

Each subject performed three different trials in order that the mean of the maximal effort of the target muscle could be determined. To evaluate MVC for the TrA, all the subjects were asked to be in the crook-lying position with flexed knees, flat feet, and hips flexed to 70° (as measured with a goniometer). Then, they were instructed to hollow in and elevate their umbilicus toward the spine and maintain this position for 5 seconds. The exercise performance was closely monitored to ensure that the subjects were not tilting the pelvis backward or inhaling and elevating the rib cage to make the abdomen look flat.

The subjects performed three successive trials of each exercise with a short rest of approximately one minute between each trial to prevent fatigue. In order to measure the MVC of IO muscles in the sitting position, the hips and the chest were fixed with two straps and the subjects were asked to produce maximal rotation without flexion toward right and left sides. Three trials of this exercise were subsequently performed. A pause of one minute was allowed between the trials.^15^

**Figure 1:**
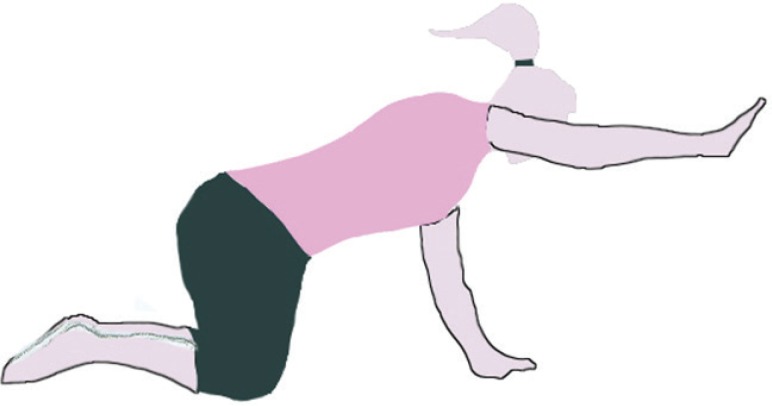
Right arm extension

**Figure 2:**
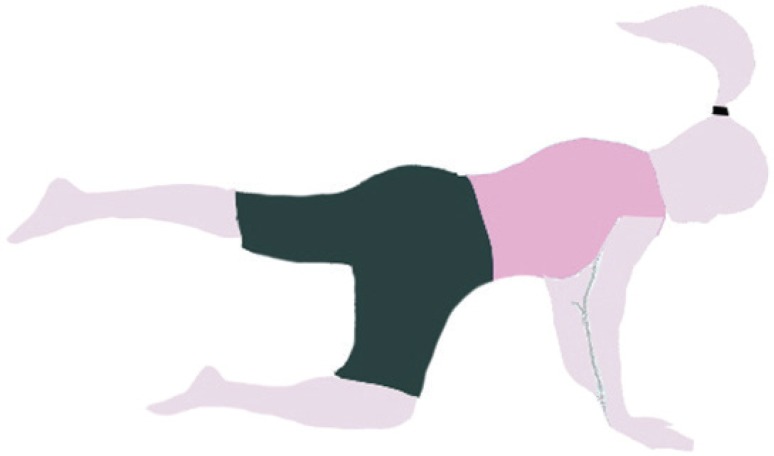
Left leg extension

**Figure 3:**
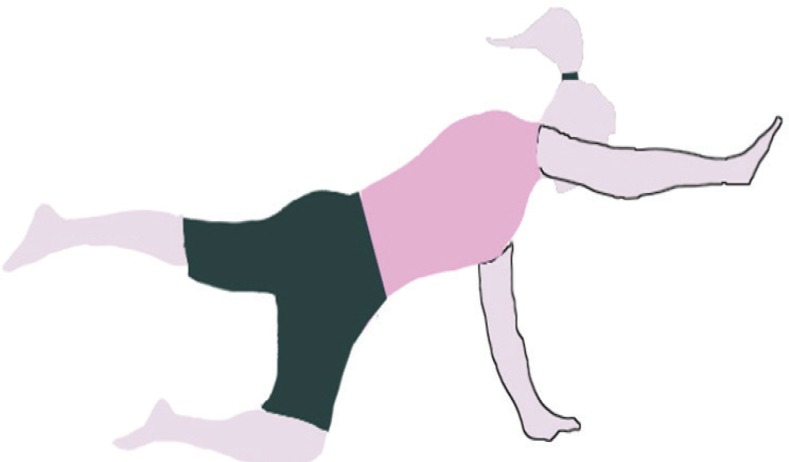
Bird-dog position

The MVC of multifidus muscles in the prone position was determined by fixing the lower extremities and the chest with two straps. The subjects performed maximal trunk extension against resistance three times. A one-minute pause was also allowed between the trials.^21^ After the measurement of MVC, all the subjects performed the four-point kneeling exercise. Correct performance of the exercise was ensured by providing the subjects with appropriate training in two sessions. These exercises were performed in the quadruped position, with the movements of the extremities being executed in a random sequence. At the beginning of each exercise, a neutral spine position was demonstrated by the examiner and the subjects were encouraged to hold this position during the whole course of the exercise. The neutral position was set about halfway between full extension and a flat position of the spine. In the right-arm extension stage, the subjects contracted their TrA muscles, maintained this contraction, and lifted the right upper extremity to a horizontal level for 5 seconds and then three trials were performed. A 15-second pause was also allowed between the trials (figure 1). In the next stage, the subjects contracted their TrA muscles and extended the left lower extremity to a horizontal level. During the static phase, the leg was held in the extended position for 5 seconds (figure 2). Afterwards, left leg extension was coupled with simultaneous rising of the right arm to the horizontal level (bird-dog); and during the static phase, this position was held for 5 seconds and registration was done (figure 3).

In this study, a band-pass filter of 10-1000 Hz and manual muscle testing were used to confirm the sites of the electrodes and eliminate the cross-talk resulting from the other muscles. Only right-handed subjects were recruited in the present study because data analysis from both sides was difficult. The right hand and the left leg were selected for motion analysis.

*Data Analysis*

For EMG amplitude analysis, artifact-free, raw EMG sections were employed. The recorded data were fully rectified and smoothed. For each of the muscles, root-mean-square amplitudes were calculated for the normalization trials using a computer algorithm which determined 500 consecutive samples (0.5 second) of raw EMG within 5 seconds. Mean MVC was used to provide a basis for EMG signal amplitude normalization. Mean normalized EMG values were calculated. During the test trials, a computer program separated the two phases based on an event marker. Also, the root-mean-square for the static and dynamic phases was calculated separately. 

*Statistical Analysis*

All the statistical analyses were performed using the SPSS statistical software (version 16), and α=0.05 was considered the significance level. The repeated measure ANOVA test with Bonferroni correction was utilized to compare the three stages of the four-point kneeling exercise. Moreover, the reliability and validity of the data were confirmed through a pilot study. A reliability study was performed to test the repeatability of the data; intraclass correlation coefficients (ICCs) were 0.66–0.87, which was indicative of good to high reliability.

## Results

In this study, the participants’ mean age, height, weight (±SD) were 23.13±2.41 years, 163.8±5.42 cm, and 54.3±8.38 kg, respectively.

The repeated measure ANOVA test for comparing the three stages of the four-point kneeling exercise showed significant differences in the muscle activation of left IO (P=0.004), left TrA (P=0.013), right multifidus (P=0.002), and left multifidus (P<0.001) muscles. The post-hoc tests with Bonferroni correction revealed that for left IO, right mulitifudus, and left mulitifudus muscles, muscle activation in the bird-dog position was higher than that of the other two exercises, while left TrA muscle activation was higher in left leg extension than in right arm extension.

Comparison between the different muscles in each stage of the four-point kneeling exercise showed that muscle activation was significantly different in all the exercises (P<0.001). The post-hoc test with Bonferroni correction revealed that in each exercise, the right TrA had the highest activation of all the muscles, whereas right and left multifidus muscles exhibited the lowest activation pattern (table 1).

**Table 1:** Comparison of mean muscle activation between the different levels of the four-point kneeling exercise

	**RT arm extension (mv)**	**LT leg extension (mv) **	**Bird-dog (mv) **	**P value**
Rt IO	1.23±0.62	0.69±1.27	0.80±1.35	0.459
Lt IO	1.51±1.29	1.36±1.76	1.63±1.90	0.004*
Rt TrA	2.14±2.57	1.75±2.58	2.63±3.11	0.053
Lt TrA	1.38±1.60	1.69±2.08	1.55±1.95	0.013*
Rt MF	0.55±0.31	0.51±0.55	0.52±0.76	0.002*
Lt MF	0.85±0.48	0.35±0.64	0.86±1.01	<0.001*
P value	<0.001*	<0.001*	<0.001*	

IO: Internal oblique; TrA: Transversus abdominis; MF: Multifidus; Rt: Right; Lt: Left; N=30, *Statistically significant

According to the results, statistically significant differences (P<0.05) were found in the activation of all the muscles, except for the right IO. On the other hand, the amplitudes of these muscles for the bird-dog position were significantly higher than those recorded for right arm extension. 

Furthermore, a statistically significant difference was found between left leg extension and bird-dog position (P<0.05) in as much as the left multifidus was activated at a significantly higher level than the other muscles in the bird-dog stage. 

## Discussion

The current study aimed to compare the EMG amplitudes of trunk and lumbar muscles during the performance of the three stages of the four-point kneeling position. The results showed that the mean activation of abdominal and lumbar muscles was different in the three stages of the four-point kneeling exercise. Overall, the right TrA had the highest activation of all the muscles, while right and left multifidus muscles showed the lowest activation pattern. This finding is related to the role of the TrA in every trunk and limb movement.

In order to provide spinal stability, the central nervous system (CNS) estimates the amount of disturbance produced by the motion of the limbs and sends the inputs to the TrA proprioceptive receptors, which trigger coordinated muscle activation. Therefore, the feed-forward mechanism is performed by the CNS in two ways: (1) non-directional for the excitation of intrinsic muscles and (2) direction-specific for the control of spinal situations.^4^

The TrA is a primary trunk stabilizer via the modulation of intra-abdominal pressure, tension through the thoracolumbar fascia, and compression of sacroiliac joints. Richardson et al.^4^ demonstrated that a voluntary contraction of the TrA reduced the laxity of the sacroiliac joint. Another study showed different levels of the feed-forward contraction of the TrA during rapid arm movements.^22^

The findings of the present study revealed that the activation of left IO and right and left mulitifudus muscles in the bird-dog position was higher than that in the other two exercises. The study results also showed that the left TrA activation in left leg extension was higher than that in right arm extension. In general, IO and TrA muscles have anatomical similarities in that both are among the muscles comprising the structure of the lower abdominal wall and they have similar functions. However, unlike the TrA, the IO affects the spine because of its direction.^4^ The IO attaches to the posterior layer of the thoracolumbar fascia. Contraction of this muscle creates a lateral tension force on the thoracolumbar fascia, which creates the intrinsic translational and rotational stabilization of the spinal unit.^20^ This should be a reason for the higher activation of this muscle during the bird-dog position. Also, Huang^23^ stated that the unilateral multifidus activation increased in the bird-dog position. Because the body weight is supported by one knee and elbow in the bird-dog position, which is an unstable status, the torque of the arm and leg is greater and the multifidus muscle shows a higher activation potential. On the other hand, lumbar multifidus and erector spine muscles have a relatively high proportion of type 1 (slow twitch) muscle fibers, which makes them well-suited for endurance or sustaining contraction activities. During the bird-dog exercise, these muscles produce EMG signal amplitudes of 29% maximal isometric voluntary contraction (MIVC).^23^ According to Arokoski,^16^ in the bird-dog exercise with weights on the hand, unilateral leg extension, and unbalanced limb movements, there is an increase in the trunk muscle activation, which contributes to the preservation of the spinal stability. The increase in the activation of left IO and left and right multifidus muscles in the bird-dog exercise may be related to the unstable body position. Accordingly, the core muscles are recruited for proprioception, balance, and energy transfer from the lower extremity to the upper extremity and hold a neutral abdominal posture.^24^

In the present study, the left TrA activation in left leg extension was higher than that in right arm extension. Callaghan reported that in order to reduce loads on the spine in exercises such as leg extension with the spine being held isometrically, the trunk muscles exhibited asymmetrical activity.^25^ In single leg extension, while the subject is on the hands and knees, he/she produces mild extensor activity and lower spine compression less than 2005 N. Raising the contralateral arm (bird-dog) increases extensor muscle activation and also spine compression to more than 3000 N. Sufficient stability is ensured with mild abdominal bracing.^26^ According to a recent study, the reciprocal index of the TrA depended on the magnitude and direction of perturbation, refuting the “corset hypothesis” as a normal mechanism for the TrA prior to arm movement. Therefore, the feed-forward activity of the TrA is asymmetric and depends on the direction of perturbation due to arm movement.^22^

Based on this study, muscle activation increased in the different stages of the four-point kneeling exercise, and the TrA activation was dominant in this position. Since the stabilization exercises exerted a minimal load on the spinal column, it can be regarded as an important clinical approach to curing acute LBP in rehabilitation centers. These exercises are used to strengthen the muscles affecting the spinal stabilization system. In addition, the present study demonstrated that the muscle activation in arm extension was less than that in the bird-dog position. As was mentioned before, in the bird-dog position, the body is unstable and patients with weak muscles are not able to tolerate this exercise. Thus, arm extension, which has not been investigated in the previous studies, can be deemed the starting stage of quadruped exercises before progress to the bird-dog exercise. Oliveira^27^ also suggested the progression of exercises from simple to complex because of the high demands of muscle activation during complex exercises. For the all the research hitherto reported in the existing literature, comparison should be made between LBP patients and healthy individuals to shed further light on the subject.

## Conclusion

In the four-point kneeling position performed by our healthy subjects, all the muscles responsible for core stability seemed to work in harmony. However, in the different positions, as loads were applied to the spine and the bases of support were changed, muscle activation was altered in order to balance the stability and demands of the movements.
